# Cyclo­hexyl­ammonium 4-meth­oxy­benzoate

**DOI:** 10.1107/S1600536811027127

**Published:** 2011-07-30

**Authors:** Bin Wei

**Affiliations:** a Ordered Matter Science Research Center, Southeast University, Nanjing 210096, People’s Republic of China

## Abstract

In the crystal of the title molecular salt, C_6_H_14_N^+^·C_8_H_7_O_3_^−^, strong N—H⋯O hydrogen bonds are formed between the ammonium H atoms and the carboxyl­ate O atoms. The resulting supra­molecular structure is based on chains running in the [010] direction. The dihedral angle between the –CO_2_ group and the benzene ring is 8.94 (17)° and the methoxy C atom deviates by 1.374 Å from the ring.

## Related literature

The title compound was studied during our search for aromatic compounds containing ammonium salts or amidogens having dielectric–ferroelectric properties (Wu *et al.*, 2011 ▶). For general background on ferroelectric metal-organic frameworks, see: Ye *et al.* (2006 ▶); Zhang *et al.* (2008 ▶, 2010 ▶); Fu *et al.* (2009 ▶). 
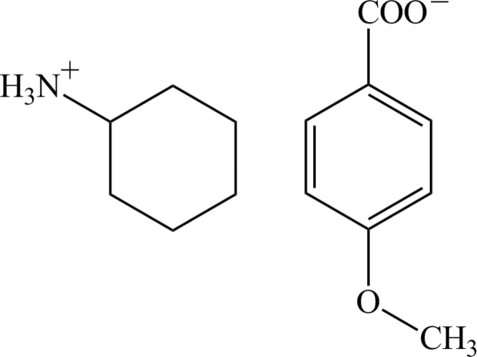


## Experimental

### 

#### Crystal data

C_6_H_14_N^+^·C_8_H_7_O_3_^−^
*M* *_r_* = 251.32
Monoclinic, 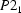

*a* = 8.9076 (18) Å
*b* = 6.6025 (13) Å
*c* = 11.778 (2) Å
β = 102.85 (3)°
*V* = 675.3 (2) Å^3^
*Z* = 2
Mo *K*α radiation
μ = 0.09 mm^−1^
*T* = 293 K
0.2 × 0.2 × 0.2 mm

#### Data collection

Rigaku Mercury CCD diffractometer
Absorption correction: multi-scan (*CrystalClear*; Rigaku, 2005 ▶) *T*_min_ = 0.842, *T*_max_ = 1.000
7050 measured reflections
1685 independent reflections
1460 reflections with *I* > 2σ(*I*)
*R*_int_ = 0.030

#### Refinement

*R*[*F* ^2^ > 2σ(*F* ^2^)] = 0.041
*wR*(*F* ^2^) = 0.095
*S* = 1.08
1685 reflections
165 parameters
1 restraint
H-atom parameters constrained
Δρ_max_ = 0.14 e Å^−3^
Δρ_min_ = −0.18 e Å^−3^

### 

Data collection: *CrystalClear* (Rigaku, 2005 ▶); cell refinement: *CrystalClear*; data reduction: *CrystalClear*; program(s) used to solve structure: *SHELXS97* (Sheldrick, 2008 ▶); program(s) used to refine structure: *SHELXL97* (Sheldrick, 2008 ▶); molecular graphics: *SHELXTL* (Sheldrick, 2008 ▶); software used to prepare material for publication: *SHELXTL*.

## Supplementary Material

Crystal structure: contains datablock(s) I, global. DOI: 10.1107/S1600536811027127/bh2364sup1.cif

Structure factors: contains datablock(s) I. DOI: 10.1107/S1600536811027127/bh2364Isup2.hkl

Supplementary material file. DOI: 10.1107/S1600536811027127/bh2364Isup3.cml

Additional supplementary materials:  crystallographic information; 3D view; checkCIF report

## Figures and Tables

**Table 1:** Hydrogen-bond geometry (Å, °)

*D*—H⋯*A*	*D*—H	H⋯*A*	*D*⋯*A*	*D*—H⋯*A*
N1—H1*C*⋯O1	0.89	1.86	2.744 (3)	173
N1—H1*A*⋯O2^i^	0.89	1.91	2.787 (2)	167
N1—H1*B*⋯O2^ii^	0.89	1.95	2.830 (3)	168

Symmetry codes: (i) 
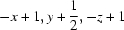
; (ii) 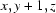.

**Fig. 1.**
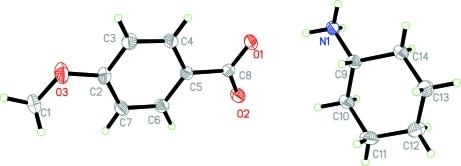
The molecular structure of the title compound, with displacement ellipsoids at the 30% probability level.

**Fig. 2.**
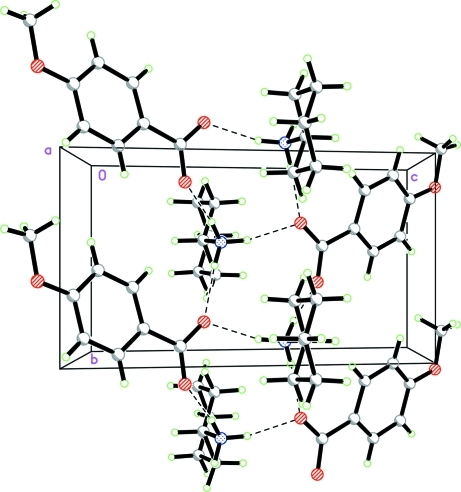
A view of the packing of the title compound, along the a axis. Dashed lines indicate hydrogen bonds.

## supplementary crystallographic information

### Comment

Our research deals with new dielectric-ferroelectric materials. Recent studies have revealed that organic salt compounds which have one or more amidogens probably have this kind of property (Fu *et al.*, 2009; Zhang *et al.*, 2008, 2010; Ye *et al.*, 2006). Thus, we are searching for aromatic compounds containing amidogens having dielectric-ferroelectric properties (Wu *et al.*, 2011). Unfortunately, the dielectric constant of the title compound as a function of temperature indicates that the permittivity is basically temperature-independent below the melting point of the salt (413 K – 415 K). We have found that cyclohexylammonium 4-methoxybenzoate has no dielectric inhomogeneity from 80 K to 405 K. Herein, we describe the crystal structure of this compound.

The asymmetric unit of the title compound consists of a cyclohexylammonium cation, and a 4-methoxybenzoate anion (Fig. 1). Strong N—H···O hydrogen bonds are formed between the H atoms of the ammonium group and the O atoms of the carboxylate group, which also make great contribution to the stability of the crystal structure, linking the cations and anions into chains along the *b* axis (Table 1 and Fig. 2).

### Experimental

The title compound was obtained by addition of *para*-methoxybenzoic acid (1.52 g, 0.01 mol) to a solution of cyclohexylamine (1.02 g, 0.01 mol) in methanol, in the stoichiometric ratio 1:1. Good quality single crystals were obtained by slow evaporation after two days (the chemical yield is 45%).

### Refinement

All H atoms attached to C atoms were fixed geometrically and treated as riding with C—H = 0.97 Å (methylene), C—H = 0.96 Å (methyl), C—H = 0.98 Å (methine), and C—H = 0.93 Å (aromatic), and with *U*_iso_(H) = 1.2*U*_eq_(C except methyl) or *U*_iso_(H) = 1.5*U*_eq_(C of methyl). The H atoms bonded to N1 were refined as riding atoms with N—H = 0.89 Å, and *U*_iso_(H) = 1.5*U*_eq_(N1). Since no significant anomalous dispersion is expected for this formula, measured Friedel pairs (1408) were merged.

### Crystal data

**Table:**

C_6_H_14_N^+^·C_8_H_7_O_3_^−^	*F*(000) = 272
*M**_r_* = 251.32	*D*_x_ = 1.236 Mg m^−^^3^
Monoclinic, *P*2_1_	Melting point: 413 K
Hall symbol: P 2yb	Mo *K*α radiation, λ = 0.71073 Å
*a* = 8.9076 (18) Å	θ = 6.2–55.3°
*b* = 6.6025 (13) Å	µ = 0.09 mm^−^^1^
*c* = 11.778 (2) Å	*T* = 293 K
β = 102.85 (3)°	Prism, colourless
*V* = 675.3 (2) Å^3^	0.2 × 0.2 × 0.2 mm
*Z* = 2	

### Data collection

**Table:**

Rigaku Mercury CCD diffractometer	1685 independent reflections
Radiation source: fine-focus sealed tube	1460 reflections with *I* > 2σ(*I*)
graphite	*R*_int_ = 0.030
Detector resolution: 28.5714 pixels mm^-1^	θ_max_ = 27.5°, θ_min_ = 3.2°
ω scans	*h* = −11→11
Absorption correction: multi-scan (*CrystalClear*; Rigaku, 2005)	*k* = −8→8
*T*_min_ = 0.842, *T*_max_ = 1.000	*l* = −15→15
7050 measured reflections	

### Refinement

**Table:**

Refinement on *F*^2^	Primary atom site location: structure-invariant direct methods
Least-squares matrix: full	Secondary atom site location: difference Fourier map
*R*[*F*^2^ > 2σ(*F*^2^)] = 0.041	Hydrogen site location: inferred from neighbouring sites
*wR*(*F*^2^) = 0.095	H-atom parameters constrained
*S* = 1.08	*w* = 1/[σ^2^(*F*_o_^2^) + (0.0414*P*)^2^ + 0.1056*P*] where *P* = (*F*_o_^2^ + 2*F*_c_^2^)/3
1685 reflections	(Δ/σ)_max_ < 0.001
165 parameters	Δρ_max_ = 0.14 e Å^−^^3^
1 restraint	Δρ_min_ = −0.18 e Å^−^^3^
0 constraints	

### Fractional atomic coordinates and isotropic or equivalent isotropic displacement parameters (Å^2^)

**Table:**

	*x*	*y*	*z*	*U*_iso_*/*U*_eq_	
C1	0.1890 (3)	−0.1036 (5)	1.0959 (2)	0.0572 (7)	
H1D	0.1425	−0.1725	1.0250	0.086*	
H1E	0.1322	−0.1329	1.1543	0.086*	
H1F	0.2934	−0.1488	1.1222	0.086*	
C2	0.2544 (3)	0.1768 (4)	0.9883 (2)	0.0408 (6)	
C3	0.2302 (3)	0.3798 (4)	0.9597 (2)	0.0459 (6)	
H3	0.1750	0.4605	1.0007	0.055*	
C4	0.2879 (3)	0.4619 (3)	0.8707 (2)	0.0390 (5)	
H4	0.2723	0.5987	0.8530	0.047*	
C5	0.3689 (2)	0.3440 (3)	0.80676 (18)	0.0320 (5)	
C6	0.3940 (3)	0.1422 (4)	0.83805 (19)	0.0381 (5)	
H6	0.4490	0.0612	0.7971	0.046*	
C7	0.3391 (3)	0.0583 (4)	0.9290 (2)	0.0420 (6)	
H7	0.3592	−0.0766	0.9498	0.050*	
C8	0.4250 (2)	0.4355 (3)	0.70654 (19)	0.0338 (5)	
C9	0.7266 (2)	0.9048 (4)	0.64892 (18)	0.0352 (5)	
H9	0.7382	0.9081	0.7336	0.042*	
C10	0.7925 (3)	0.7064 (4)	0.6172 (3)	0.0476 (6)	
H10A	0.7763	0.6960	0.5332	0.057*	
H10B	0.7396	0.5943	0.6445	0.057*	
C11	0.9646 (3)	0.6941 (4)	0.6720 (3)	0.0562 (7)	
H11A	0.9796	0.6892	0.7561	0.067*	
H11B	1.0061	0.5703	0.6468	0.067*	
C12	1.0512 (3)	0.8741 (5)	0.6383 (2)	0.0534 (7)	
H12A	1.1587	0.8656	0.6782	0.064*	
H12B	1.0458	0.8710	0.5552	0.064*	
C13	0.9841 (3)	1.0711 (4)	0.6698 (3)	0.0533 (7)	
H13A	1.0376	1.1837	0.6435	0.064*	
H13B	0.9994	1.0805	0.7538	0.064*	
C14	0.8118 (3)	1.0854 (4)	0.6142 (2)	0.0435 (6)	
H14A	0.7704	1.2094	0.6391	0.052*	
H14B	0.7970	1.0896	0.5301	0.052*	
N1	0.5590 (2)	0.9176 (3)	0.59367 (15)	0.0354 (4)	
H1A	0.5457	0.9073	0.5167	0.053*	
H1B	0.5221	1.0358	0.6114	0.053*	
H1C	0.5093	0.8172	0.6199	0.053*	
O1	0.4131 (2)	0.6223 (3)	0.69286 (16)	0.0517 (5)	
O2	0.47886 (19)	0.3187 (3)	0.64103 (13)	0.0452 (4)	
O3	0.1875 (2)	0.1079 (3)	1.07544 (16)	0.0599 (5)	

### Atomic displacement parameters (Å^2^)

**Table:**

	*U*^11^	*U*^22^	*U*^33^	*U*^12^	*U*^13^	*U*^23^
C1	0.0654 (16)	0.0534 (16)	0.0579 (16)	−0.0038 (15)	0.0249 (13)	0.0187 (15)
C2	0.0483 (13)	0.0402 (13)	0.0366 (12)	−0.0055 (11)	0.0155 (10)	0.0006 (10)
C3	0.0602 (15)	0.0351 (12)	0.0500 (14)	−0.0010 (12)	0.0284 (11)	−0.0100 (11)
C4	0.0459 (13)	0.0288 (11)	0.0453 (13)	0.0011 (10)	0.0162 (10)	−0.0006 (9)
C5	0.0312 (10)	0.0313 (12)	0.0329 (10)	−0.0017 (9)	0.0058 (8)	−0.0004 (9)
C6	0.0429 (12)	0.0343 (12)	0.0392 (12)	0.0056 (10)	0.0138 (9)	0.0000 (10)
C7	0.0534 (14)	0.0319 (12)	0.0426 (13)	0.0034 (11)	0.0148 (11)	0.0049 (10)
C8	0.0316 (10)	0.0354 (13)	0.0351 (11)	0.0018 (9)	0.0089 (8)	0.0038 (9)
C9	0.0406 (11)	0.0329 (11)	0.0327 (11)	0.0030 (10)	0.0096 (8)	0.0015 (9)
C10	0.0499 (15)	0.0259 (12)	0.0675 (18)	0.0038 (10)	0.0138 (13)	−0.0009 (11)
C11	0.0535 (16)	0.0412 (15)	0.0723 (19)	0.0159 (13)	0.0103 (14)	0.0038 (13)
C12	0.0419 (13)	0.0561 (18)	0.0619 (16)	0.0085 (13)	0.0111 (11)	−0.0013 (14)
C13	0.0448 (14)	0.0416 (16)	0.0712 (18)	−0.0015 (12)	0.0085 (13)	−0.0071 (13)
C14	0.0449 (14)	0.0298 (12)	0.0561 (15)	0.0005 (10)	0.0115 (12)	−0.0008 (11)
N1	0.0432 (10)	0.0296 (9)	0.0365 (9)	0.0016 (8)	0.0155 (7)	0.0016 (8)
O1	0.0631 (11)	0.0335 (9)	0.0680 (12)	0.0048 (9)	0.0350 (9)	0.0120 (9)
O2	0.0629 (11)	0.0387 (9)	0.0398 (9)	0.0088 (8)	0.0241 (8)	0.0046 (8)
O3	0.0912 (14)	0.0467 (11)	0.0552 (11)	−0.0064 (11)	0.0446 (10)	0.0020 (9)

### Geometric parameters (Å, °)

**Table:**

C1—O3	1.417 (3)	C9—C14	1.518 (3)
C1—H1D	0.9600	C9—H9	0.9800
C1—H1E	0.9600	C10—C11	1.528 (4)
C1—H1F	0.9600	C10—H10A	0.9700
C2—O3	1.374 (3)	C10—H10B	0.9700
C2—C7	1.380 (3)	C11—C12	1.517 (4)
C2—C3	1.387 (4)	C11—H11A	0.9700
C3—C4	1.376 (3)	C11—H11B	0.9700
C3—H3	0.9300	C12—C13	1.511 (4)
C4—C5	1.392 (3)	C12—H12A	0.9700
C4—H4	0.9300	C12—H12B	0.9700
C5—C6	1.387 (3)	C13—C14	1.532 (4)
C5—C8	1.507 (3)	C13—H13A	0.9700
C6—C7	1.389 (3)	C13—H13B	0.9700
C6—H6	0.9300	C14—H14A	0.9700
C7—H7	0.9300	C14—H14B	0.9700
C8—O1	1.246 (3)	N1—H1A	0.8900
C8—O2	1.259 (3)	N1—H1B	0.8900
C9—N1	1.492 (3)	N1—H1C	0.8900
C9—C10	1.516 (3)		
			
O3—C1—H1D	109.5	C11—C10—H10A	109.6
O3—C1—H1E	109.5	C9—C10—H10B	109.6
H1D—C1—H1E	109.5	C11—C10—H10B	109.6
O3—C1—H1F	109.5	H10A—C10—H10B	108.1
H1D—C1—H1F	109.5	C12—C11—C10	111.6 (2)
H1E—C1—H1F	109.5	C12—C11—H11A	109.3
O3—C2—C7	124.6 (2)	C10—C11—H11A	109.3
O3—C2—C3	115.6 (2)	C12—C11—H11B	109.3
C7—C2—C3	119.9 (2)	C10—C11—H11B	109.3
C4—C3—C2	120.1 (2)	H11A—C11—H11B	108.0
C4—C3—H3	119.9	C13—C12—C11	111.0 (2)
C2—C3—H3	119.9	C13—C12—H12A	109.4
C3—C4—C5	121.2 (2)	C11—C12—H12A	109.4
C3—C4—H4	119.4	C13—C12—H12B	109.4
C5—C4—H4	119.4	C11—C12—H12B	109.4
C6—C5—C4	117.8 (2)	H12A—C12—H12B	108.0
C6—C5—C8	122.08 (19)	C12—C13—C14	111.2 (2)
C4—C5—C8	120.14 (19)	C12—C13—H13A	109.4
C5—C6—C7	121.7 (2)	C14—C13—H13A	109.4
C5—C6—H6	119.2	C12—C13—H13B	109.4
C7—C6—H6	119.2	C14—C13—H13B	109.4
C2—C7—C6	119.4 (2)	H13A—C13—H13B	108.0
C2—C7—H7	120.3	C9—C14—C13	110.5 (2)
C6—C7—H7	120.3	C9—C14—H14A	109.6
O1—C8—O2	124.1 (2)	C13—C14—H14A	109.6
O1—C8—C5	117.7 (2)	C9—C14—H14B	109.6
O2—C8—C5	118.20 (19)	C13—C14—H14B	109.6
N1—C9—C10	110.26 (19)	H14A—C14—H14B	108.1
N1—C9—C14	110.48 (18)	C9—N1—H1A	109.5
C10—C9—C14	111.59 (17)	C9—N1—H1B	109.5
N1—C9—H9	108.1	H1A—N1—H1B	109.5
C10—C9—H9	108.1	C9—N1—H1C	109.5
C14—C9—H9	108.1	H1A—N1—H1C	109.5
C9—C10—C11	110.5 (2)	H1B—N1—H1C	109.5
C9—C10—H10A	109.6	C2—O3—C1	117.6 (2)

### Hydrogen-bond geometry (Å, °)

**Table:**

*D*—H···*A*	*D*—H	H···*A*	*D*···*A*	*D*—H···*A*
N1—H1C···O1	0.89	1.86	2.744 (3)	173.
N1—H1A···O2^i^	0.89	1.91	2.787 (2)	167.
N1—H1B···O2^ii^	0.89	1.95	2.830 (3)	168.

Symmetry codes: (i) −*x*+1, *y*+1/2, −*z*+1; (ii) *x*, *y*+1, *z*.
